# Quality assurance and enrichment of biological and biomedical ontologies and terminologies

**DOI:** 10.1186/s12911-020-01342-4

**Published:** 2020-12-15

**Authors:** Ankur Agrawal, Licong Cui

**Affiliations:** 1 Department of Computer Science, Manhattan College, New York, USA; 2 School of Biomedical Informatics, University of Texas Health Science Center at Houston, Houston, TX USA

**Keywords:** Quality assurance, Auditing, Semantic enrichment, Mapping, Ontology

## Abstract

Biological and biomedical ontologies and terminologies are used to organize and store various domain-specific knowledge to provide standardization of terminology usage and to improve interoperability. The growing number of such ontologies and terminologies and their increasing adoption in clinical, research and healthcare settings call for effective and efficient quality assurance and semantic enrichment techniques of these ontologies and terminologies. In this editorial, we provide an introductory summary of nine articles included in this supplement issue for quality assurance and enrichment of biological and biomedical ontologies and terminologies. The articles cover a range of standards including SNOMED CT, National Cancer Institute Thesaurus, Unified Medical Language System, North American Association of Central Cancer Registries and OBO Foundry Ontologies.

## Background

Ontologies and terminologies provide structured and unambiguous ways of representing domain information in biology and biomedicine. Examples of such ontologies and terminologies include Gene Ontology [1], SNOMED CT [2], and National Cancer Institute Thesaurus (NCIt) [3]. They have served as knowledge sources for a wide range of biomedical applications including data integration and exchange, natural language processing, reasoning, and decision support [4, 5]. These ontologies and terminologies tend to be large and are regularly maintained through revisions and modifications in their lifecycle, which may result in ambiguity, redundancy and modeling inconsistencies. As such, quality assurance and enrichment of these ontologies and terminologies become more and more important as they impact all the downstream applications that rely on them, and thus an active research area. In [6], Zhu et al. provided a comprehensive review of early works on the auditing methods of biomedical terminologies regarding various quality factors. In [7], Amith et al. surveyed more recent quality assurance approaches for biomedical ontologies. In [8], Zheng et al. performed a thorough review of methods for auditing the Unified Medical Language System (UMLS), as well as ontology enrichment and alignment techniques. Two special issues [9, 10] have been organized and published to showcase the state of the art in auditing and quality assurance of biomedical terminologies and ontologies in 2009 and 2018, respectively.

In this special issue supplement, we aim to capture the most recent work on quality assurance and enrichment of biological and biomedical ontologies and terminologies. Articles were invited by sending out calls for papers to major listservers. A total of nine papers were accepted for publication in this special issue after going through a rigorous, single-blind review process. Each article was reviewed by 2–3 reviewers. The reviewers included both the authors who submitted their work as well as other prominent researchers from this field. All the reviewers had extensive experience working in the field of quality assurance and enrichment of medical terminologies. The entire process from sending out the first call for papers to the final publication of articles took approximately eleven months.

## Summary of papers in this special issue

In the paper “Missing lateral relationships in top-level concepts of an ontology” [11], the authors leverage their previous work on two types of abstraction networks, “area taxonomy” and “subtaxonomy,” to audit non-hierarchical or lateral relationships. In this study, the authors focus on grouping high-level concepts, called “top areas,” in the NCIt’s *Biological Process* hierarchy and SNOMED CT’s *Eye/vision finding* sub-hierarchy. Manual reviews by domain experts revealed missing lateral relationships, which were further used to validate their hypotheses that top areas with a large number of concepts and concepts in the deeper hierarchical depth of top areas have a high likelihood of containing missing relationships. As the authors stated, their methodology can be seen as a useful addition to the quality assurance tools available to ontology maintenance personnel.

The paper “Extending import detection algorithms for concept import from two to three biomedical terminologies” [12] introduces a novel topological pattern, called “fire ladder,” to structurally compare three terminologies (two source terminologies and one target terminology) and detect candidate concepts from the source terminologies that could potentially be imported into the target terminology. The authors explored the fire ladder patterns in ten selected terminologies in the UMLS (2018 AB release) and identified a total of 55 candidate instances for concept import, among which 39 were agreed by two domain experts and 48 by at least one expert. This is an important work on concept enrichment by leveraging external terminologies, and it may further help in enhancing semantic harmonization among different terminologies.

In “Web-based interactive mapping from data dictionaries to ontologies, with an application to cancer registry” [13], the authors present an interactive web-based tool to map data dictionary elements to ontology concepts. This tool has a recommendation engine at its core that provides a list of recommended concepts from the target ontology for an unmapped data element from the source data dictionary. This recommendation is based on a fuzzy matching algorithm. A pilot-test of the mapping between North American Association of Central Cancer Registries (NAACCR) elements extracted from Kentucky Cancer Registry (KCR) and NCIt concepts showed 47 of the 301 data elements were mapped to NCIt concepts. Of these 47, 25 were found to be correct when manually verified by domain experts. The study is important as such mapping techniques can provide semantic enrichment and interoperability between data dictionaries and ontologies.

The paper “Detecting missing IS-A relations in the NCI Thesaurus using an enhanced hybrid approach” [14] presents a structural-lexical-based methodology to identify potentially missing IS-A relationships in NCIt by using lexical features and role definitions of biomedical concept names. Missing IS-A relationships can result in erroneous output by applications that rely on NCIt as their underlying vocabulary. The authors explain this with an example where someone is searching for patients with “Cystic Neoplasm” using an NCIt powered search engine. However, “Dermoid Cyst” is currently not listed as one of the descendants (i.e., a missing IS-A relation) of “Cystic Neoplasm”. As a result, patients with “Dermoid Cyst” will be missing from the search result. The proposed method involves computing non-lattice subgraphs and identifying candidate pairs of concepts that are currently not linked by IS-A relations, modeling concepts utilizing role definitions and lexical features to represent the meaning of concepts, and performing subsumption checking for candidate pairs of concepts.The authors applied their approach to the 19.08d version of NCIt and their algorithm found 55 potentially missing IS-A relationships. Domain experts from NCI Enterprise Vocabulary Services confirmed 29 of the 55 suggested anomalies as valid and were implemented in the newer versions of the thesaurus. NCIt is widely used as a reference terminology in cancer related research and in clinical care, and studies such as this can prove to be a useful tool to improve the quality of NCIt.

In “Friend of a Friend with Benefits Ontology (FOAF+): Extending a Social Network Ontology for Public Health” [15], the authors build a social network-related ontology for use in the field of public health to logically infer dyadic social networks between individuals. This ontology which the authors call Friend of a Friend with Benefits (FOAF+) ontology, is constructed to describe the domain of social and sexual behavior as it pertains to STI transmission between individuals. FOAF+ has 713 classes, 137 object properties, 130 data properties, and 312 instances. The authors compare FOAF+ with VIVO and FOAF using semiotic metrics produced by their automated tool OntoKeeper and their evaluation found the tool to be adequate as a prototype release. Social network ontologies such as FOAF+ can prove to be helpful in aiding machines to understand and interpret social network data, to identify missing links and to discover new relational links from network data.

In “Evaluation of lexical clarification by patients reading their clinical notes: A quasi experimental interview study” [16], the authors evaluate the functionality of a patient portal at the Dutch university medical centre (UMC Utrecht) that helps patients in understanding the clinical terms in free-text medical data. A Dutch medical terminology system is used to explain the terms to the patients using synonyms and definitions. A survey of 15 participants found the functionality easy to use as well as useful, albeit with low coverage of clarification of terms. The study is important as understanding of clinical notes helps patients remember their discussion with physicians and take better care of themselves as stated by the authors in the paper.

The paper “Analysis of readability and structural accuracy in SNOMED CT” [17] presents readability and structural accuracy metrics to provide a quantitative description of the structural aspects of an ontology and possible detection of missing semantic relations in the ontology. The underlying assumption of the authors is that an ontology should be friendly for both humans and machines and the correspondence between the contents for humans and machines should provide information regarding the quality of the ontology. The authors apply their metrics to different versions of SNOMED CT to provide useful insights about its modeling and evolution over time. The study is significant as the proposed metrics can be used to improve the effectiveness of the quality assurance process by identifying areas of an ontology with low readability and structural accuracy.

In the paper “Outlier concepts auditing methodology for a large family of biomedical ontologies” [18], the authors apply an abstraction network technique called “partial-area taxonomy” to SNOMED CT’s *Specimen* hierarchy and NCIt’s *Gene* hierarchy. They validate their hypothesis that concepts in small partial-areas of the partial-area taxonomy have statistically significantly more errors than concepts in large partial-areas, which is consistent with previous studies on four hierarchies from the same family of ontologies with outgoing lateral relationships. This further proves the scalability of the small partial-area technique to be potentially applied for auditing the larger family of biomedical ontologies in BioPortal.

In the paper “Towards semantic interoperability: finding and repairing hidden contradictions in biomedical ontologies” [19], the authors explore a technique to identify and repair unsatisfiable classes by combining ontologies from the Open Biomedical Ontologies (OBO) Foundry and the OBO ontologies. The study found 636 unsatisfiable classes in the nine OBO Foundry ontologies and over 300,000 unsatisfiable across 123 OBO ontologies. The authors also present a semi-automatic repair algorithm to identify axioms that result in these unsatisfiable classes which when removed, resolves the unsatisfiable classes. Applying this algorithm, the authors identified a small set of only 117 axioms that could be removed or modified to correct all the issues that were identified across all the ontologies. Consistency is an important key towards interoperability of ontologies and this study presents an effective approach to producing consistent and coherent ontologies.

## Conclusions

With advances in health information technologies and their widespread adoption, ontologies and terminologies in biology and biomedicine have become ever more important to capture patient data in a consistent and standardized manner and for their effective transmission and communication. This has resulted in a call for more advances and research studies in the field of quality assurance and enrichment of these ontologies and terminologies. While research in this field has gained momentum over the past decade, the guest editors believe that advances will continue towards delivering more automated techniques for quality assurance and enrichment of ontologies and terminologies.

## Data Availability

Not applicable.
